# Retinoic Acid Increases Cellular Cholesterol in Leishmania donovani-Infected Macrophages in an mTOR-Independent Manner

**DOI:** 10.1128/spectrum.02699-22

**Published:** 2022-10-20

**Authors:** Satya Prakash, Ambak Kumar Rai

**Affiliations:** a Department of Biotechnology, Motilal Nehru National Institute of Technology Allahabad, Prayagraj, Uttar Pradesh, India; University of São Paulo

**Keywords:** *Leishmania*, retinoic acid, cholesterol, mTOR

## Abstract

Infection with Leishmania donovani reduces cellular cholesterol and thus deprives the host cells by inhibiting its synthesis and uptake. Changes in cholesterol levels increase the chance of attachment and internalization of L. donovani in macrophages (Mϕ). Retinoic acid (RA), an important micronutrient, restores the lysosomal uptake of cholesterol in L. donovani-infected Mϕ. Importantly, mammalian (or mechanistic) target of rapamycin complex 1 (mTORC1) increases the cellular cholesterol level by increasing expression of sterol regulatory element-binding protein 2 (*SREBP2*). Whether the efficacy of RA in L. donovani-infected Mϕ is mediated by mTOR is not yet established. Moreover, there are contradicting reports suggesting potential activation and inhibition of mTOR in L. donovani-infected Mϕ. Intrigued by this, we attempted to understand the RA-mediated restoration of cholesterol as well as the possible roles of mTORC1, if any. Our findings suggest that L. donovani infection impairs the synthesis of 3-hydroxy-3-methylglutaryl coenzyme A reductase (HMGCR), uptake of low-density lipoprotein receptor (LDLR), and secretion of ATP-binding cassette transporter (ABCA1) in Mϕ. L. donovani infection possibly impairs mTORC1 formation, as it inhibits the expression of regulatory-associated protein of mammalian target of rapamycin (*RAPTOR*). Importantly, all these are restored upon RA supplementation. RA also restores the levels of *SREBP2* in L. donovani-infected Mϕ, resulting in increased cellular cholesterol and thus reducing the parasite burden. When mTORC1 was inhibited, RA exerted a similar response in L. donovani-infected Mϕ; i.e., it restored cholesterol levels and reduced the parasite burden. In summary, RA restores cholesterol levels in L. donovani-infected Mϕ and reduces the parasite burden in an mTOR-independent manner.

**IMPORTANCE** People who reside in regions where leishmaniasis is endemic and who lack proteins, iron, zinc, and vitamin A in their diet are more prone to develop visceral leishmaniasis (VL) as a full-blown disease. Vitamin A deficiency favors the development of a parasitic infection in the human host, and the WHO recommends administering 200,000-IU doses to VL patients on admission. Additionally, *Leishmania* entry and its survival inside the host are achieved by utilizing host cholesterol, as all trypanosomatids lack *de novo* synthesis of sterol. We have already shown that RA regulates cellular cholesterol levels associated with an efficient immune response. A deficiency of retinoic acid (RA) favors the parasite in Leishmania donovani-infected macrophages by downregulating the immune response. In the present work, we observed that RA restores cellular cholesterol levels in Leishmania donovani-infected macrophages. This study proposes using RA as an immune potentiator along with standard therapy.

## INTRODUCTION

The protozoan parasites that cause leishmaniasis, belonging to the genus *Leishmania*, are transmitted to the human host by the bite of the infected female sandfly. Worldwide, four different clinical forms of leishmaniasis exist, categorized as visceral leishmaniasis (VL), post-kala-azar dermal leishmaniasis (PKDL), cutaneous leishmaniasis (CL), and mucocutaneous leishmaniasis (MCL) (1). VL is prevalent in Brazil, China, Ethiopia, India, Iraq, Kenya, Nepal, Somalia, and Sudan and is caused by Leishmania donovani in the Indian subcontinent. If not treated promptly, it can be fatal. Drug resistance and lack of new effective drugs are challenges which need global attention. Though the number of VL cases has dropped during the last few years globally, however, 50,000 to 90,000 new cases still occur every year, and over 600 million people are at risk (2–4).

The nutritional status of people at risk plays an important role in overall immune status and in the initial response to encountering the parasite. Once the infection is established in host cells and chemotherapy begins, the immune response, which is affected by nutritional status, restricts the parasite through drug-immune interphase. Since the *Leishmania* parasite targets the malnourished population, which has overall low immunity, therapeutic advancement may not be possible unless we target malnutrition in a general or specific manner. This is possibly the reason that primary infection often progresses to full-blown disease (3), and treatment may also fail due to poor immune responses in malnourished VL patients. Our earlier findings show low levels of retinoic acid (RA) in VL patients, and an *in vitro* infection with L. donovani shows decreased endogenous conversion of RA from retinol, a dietary form of vitamin A (5). This results in poor immune responses in infected macrophages (Mϕ) showing increased expression of arginase-1 and interleukin 10 (IL-10). Our recent findings also show that RA restores the cellular cholesterol by increasing its lysosomal uptake (via NPC-1 and NPC-2 proteins) in L. donovani-infected Mϕ (6). In extrahepatic cells such as Mϕ, cellular cholesterol is maintained in three possible ways; synthesis, uptake, and efflux. Briefly, cellular synthesis from acetyl coenzyme A (acetyl-CoA) is regulated by the enzyme 3-hydroxy-3-methylglutaryl-CoA (HMG-CoA) reductase (HMGCR), and cellular uptake is mediated by low-density lipoprotein receptor (LDLR) and regulated by PCSK9. Cellular efflux of cholesterol in the form of high-density lipoprotein (HDL) is routed through ABCA1 present on the surface of Mϕ. Their status is not yet completely understood in L. donovani-infected Mϕ.

In general, it is believed that RA, an active metabolite of vitamin A, performs various biological functions, including immune response regulation through interaction with retinoic acid response element (RARE)-binding receptors. Our findings showed the presence of RARE sites upstream of *npc-1* and *npc-2* genes, which are involved in lysosomal uptake of cholesterol (6). Interestingly, we have also shown that RA exerts its effect on cellular cholesterol level in an mTORC1 (mammalian [or mechanistic] target of rapamycin complex 1)-dependent manner in Mϕ. However, not much is known about its role in L. donovani-infected Mϕ. Moreover, there are contradictory reports on the status of the mTORC1 pathway in L. donovani infection. One report suggests increased phosphorylation at Ser2148 and Ser2481 in the mTOR kinase domain in L. donovani-infected Mϕ (7). In contrast, another report suggests inhibition of mTOR activity in L. donovani-infected Mϕ (8). Thus, the status of mTOR pathway in L. donovani-infected Mϕ and its potential involvement in RA-mediated restoration of cellular cholesterol need to be revisited. In the present work, we attempted to clarify this in an L. donovani-infected mouse macrophage line.

## RESULTS

### RA consumption increased in L. donovani-infected macrophages.

**FIG 1:**
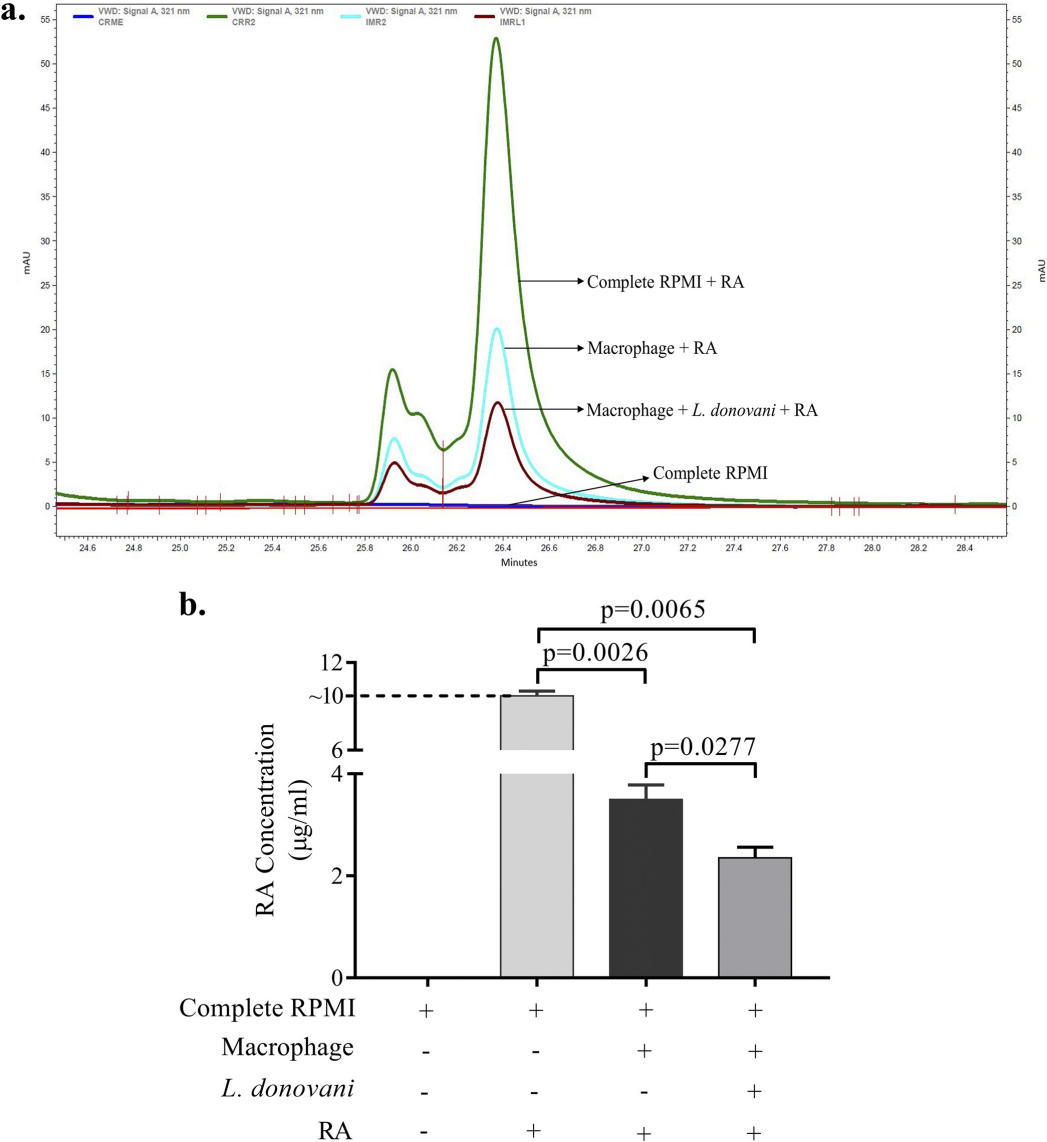
(a) HPLC chromatogram showing the residual RA in the deproteinized culture supernatants of Mϕ and Leishmania donovani-infected Mϕ. Cultures were supplemented with RA at a dose of 10 μg/mL for 12 h. (b) Bar diagram showing the residual RA under similar conditions. Medium and RA-containing medium are shown as controls. The experiments were performed in three independent sets, and each set was performed in duplicate. The difference between two conditions is considered significant if the *P* value is less than 0.05.

At first, we attempted to check whether RA supplementation is used by the L. donovani-infected Mϕ or whether cellular entry and uptake of RA in Mϕ are unaffected by L. donovani infection. Thus, we measured the levels of residual RA in the culture supernatants of uninfected and L. donovani-infected Mϕ (Fig. 1a). The cultures were supplemented with 10 μg/mL of RA. Findings showed more consumption of RA in L. donovani-infected Mϕ, suggesting intactness of cellular uptake/entry of RA (Fig. 1b).

### L. donovani infection impairs the synthesis, uptake, and efflux of cholesterol in Mϕ.

**FIG 2:**
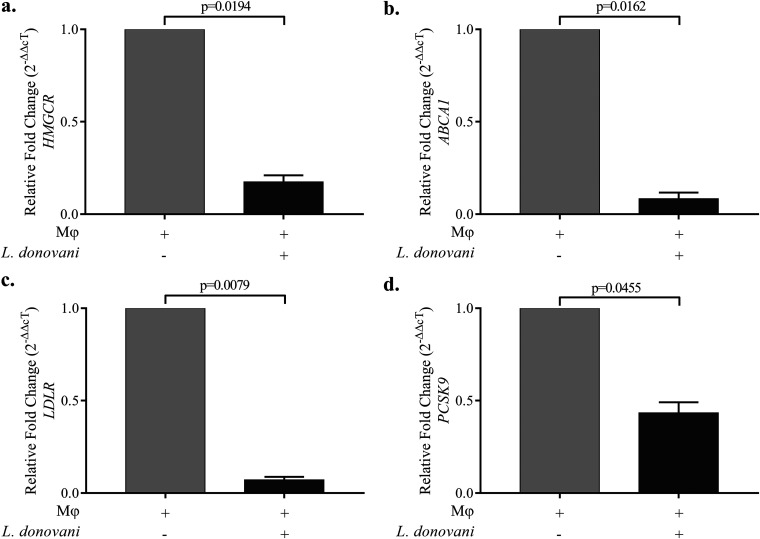
Leishmania donovani infection decreased the expression of cholesterol-regulating genes. The bar diagrams show the relative fold changes (2^−ΔΔ^*^cT^*) in mRNA expression of (a) *HMGCR*, (b) *ABCA1*, (c) *LDLR*, and (d) *PCSK9* in Mϕ only and in L. donovani-infected Mϕ. The expression in untreated Mϕ is taken as 1 to compare relative fold change under other conditions. The experiments were performed in three independent sets, and each set was performed in triplicate. The difference between two conditions is considered significant if the *P* value is ≤0.05.

We measured the expression of *HMGCR*, *ABCA1*, and *LDLR* in L. donovani-infected Mϕ in order to measure synthesis, efflux, and uptake of cholesterol, respectively. Our findings showed a significant decrease in the mRNA expression of *HMGCR* (Fig. 2a) as well as *ABCA1* (Fig. 2b) in L. donovani-infected Mϕ, suggesting impaired synthesis and efflux. The mRNA expression of *LDLR* gene was also decreased in L. donovani-infected Mϕ (Fig. 2c). Furthermore, PCSK9, a protein involved in the delivery of LDL complex to the lysosomal compartment for degradation, was also measured under similar conditions. Our findings showed decreased mRNA expression of *PCSK9* in L. donovani-infected Mϕ (Fig. 2d).

### L. donovani infection inhibits the expressions *RAPTOR* and *SREBP2* in Mϕ.

**FIG 3:**
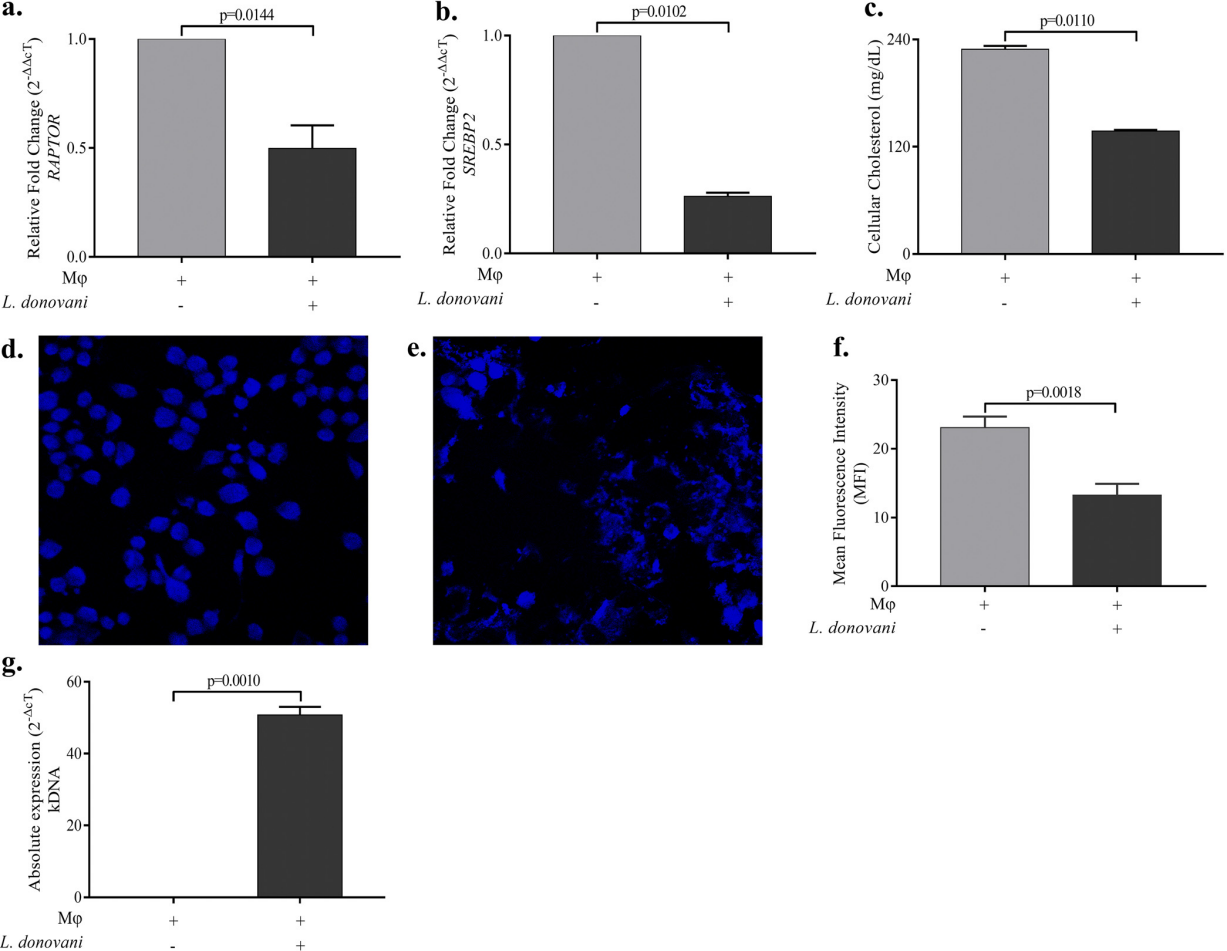
Leishmania donovani-driven inhibition of *RAPTOR* and *SREBP2* expression in infected Mϕ. (a and b) Bar diagrams showing relative fold changes (2^−ΔΔ^*^cT^*) in mRNA expression of (a) *RAPTOR* and (b) *SREBP2* in Mϕ only and in L. donovani-infected Mϕ. (c) The cellular cholesterol level was measured using biochemical methods in Mϕ only and in L. donovani-infected Mϕ. (d and e) Confocal images of filipin III-stained Mϕ only (d) and L. donovani-infected Mϕ (e). (f) Bar graph showing mean fluorescence intensity (MFI) of filipin III staining in uninfected Mϕ and L. donovani-infected Mϕ, calculated using ImageJ software. (g) Parasitic load, determined as kDNA expression in infected Mϕ. The experiments were performed in three independent sets, and each set was performed in triplicate. The difference between two conditions is considered significant if the *P* value is ≤0.05.

It is established that the mTORC1 pathway regulates the expressions of *SREBP2*. Moreover, there are contradictory reports on the status of mTOR pathways in L. donovani-infected Mϕ. Thus, we were interested to measure the mRNA expression of *RAPTOR*, which encodes a regulatory protein of mTORC1, and *SREBP2*, which encodes a cholesterol-sensing transcription factor initially sequestered in the endoplasmic reticulum and involved in expression of regulatory genes in cholesterol biosynthesis and uptake. Our findings showed a significant decrease in the mRNA expression of *RAPTOR* (Fig. 3a) and *SREBP2* (Fig. 3b) in L. donovani-infected Mϕ. Furthermore, significant depletion of cellular cholesterol was also observed in infected Mϕ through biochemical estimation (Fig. 3c). To further validate this, we showed loss of cholesterol in L. donovani-infected Mϕ through a fluorescence-based study using the dye filipin III (Fig. 3d to f). To detect the parasite burden under similar conditions, we measured kinetoplast DNA (kDNA) expression, which is used to indicate parasite load. Our finding showed significant increases in parasite loads in infected Mϕ (Fig. 3g).

### RA restores the cellular cholesterol levels in infected Mϕ and reduces the parasite load.

**FIG 4:**
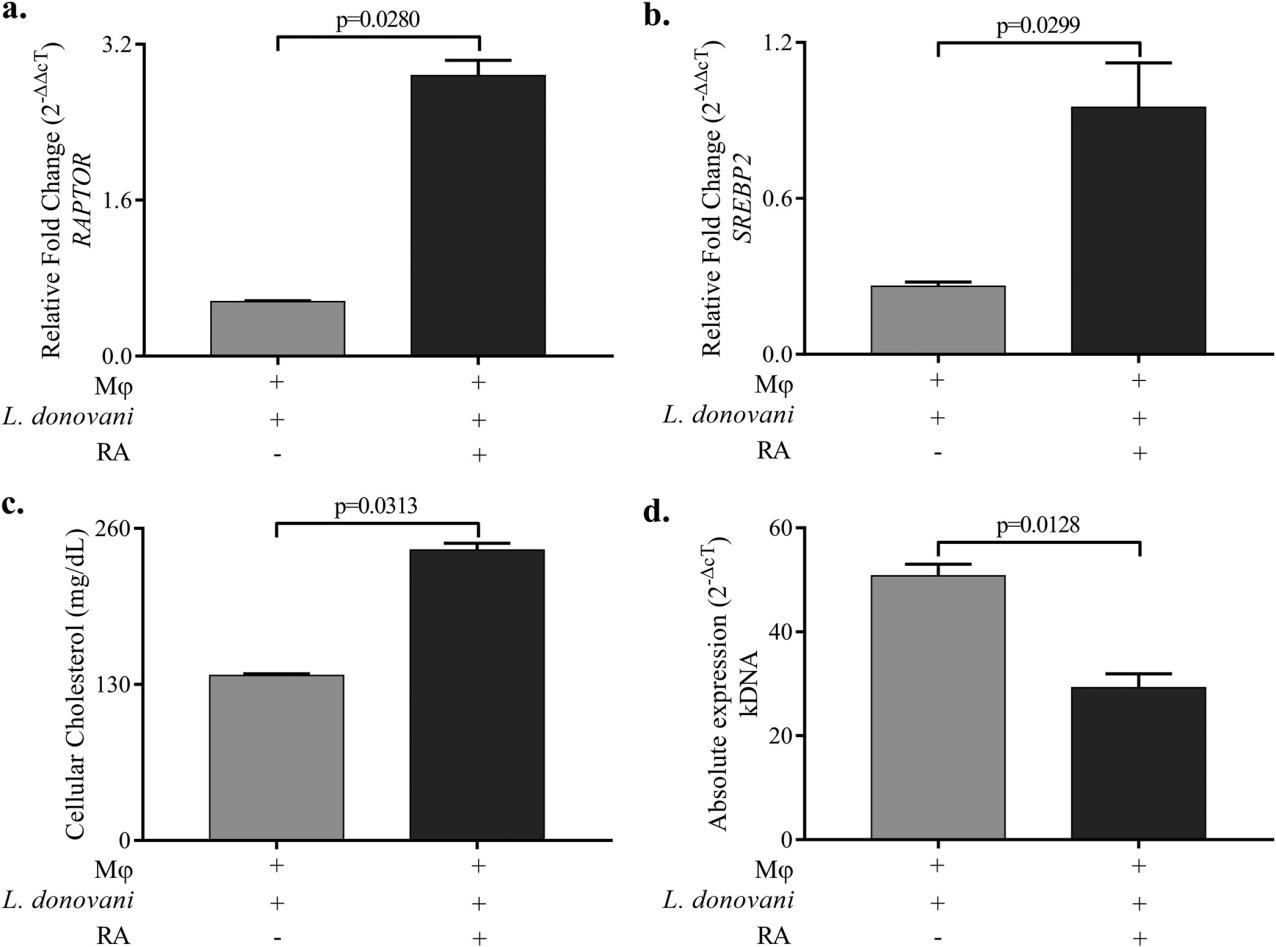
RA restores the cholesterol levels in infected Mϕ and thus reduces the parasite load. The bar graphs show the relative fold changes (2^−ΔΔ^*^cT^*) in mRNA expressions of (a) *RAPTOR* and (b) *SREBP2* in Leishmania donovani-infected Mϕ upon treatment with RA. Leishmania donovani-infected Mϕ were used as a control. (c) The levels of cellular cholesterol were estimated biochemically in L. donovani-infected Mϕ and RA-supplemented L. donovani-infected Mϕ. (d) Bar graph showing the absolute expression (2^−Δ^*^cT^*) of kDNA (i.e., parasitic load) in RA-supplemented L. donovani-infected Mϕ. L. donovani-infected Mϕ were used as a control (expression is taken as 1). The experiments were performed in three independent sets, and each set was performed in triplicate. The difference between two conditions is considered significant if the *P* value is ≤0.05.

We have already demonstrated that RA increases the mRNA expression of *npc-1* and *npc-2* genes, which are involved in lysosomal transfer of cholesterol, and restores expression in L. donovani-infected Mϕ (6). Since we observed decreases in the mRNA expression of *RAPTOR* and *SREBP2* genes in L. donovani-infected Mϕ, we measured the levels of these genes after treating the infected cells with RA. Our findings showed restoration of the expression of *RAPTOR* (Fig. 4a) and *SREBP2* (Fig. 4b) upon RA supplementation postinfection. When we supplemented infected cells with RA, we found increases in the levels of cellular cholesterol over those of untreated infected cells (Fig. 4c). Importantly, parasitic load was significantly reduced upon RA treatment of L. donovani-infected Mϕ (Fig. 4d).

### RA-driven restoration of cholesterol in L. donovani-infected Mϕ is not mediated through the mTORC1 pathway.

**FIG 5:**
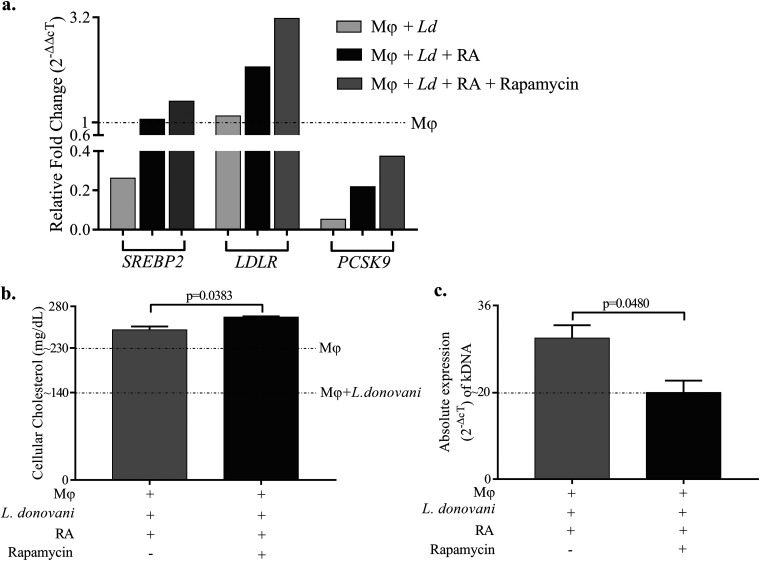
RA-driven restoration of cholesterol in Leishmania donovani-infected Mϕ is independent of the mTORC1 pathway. (a) Bar graph showing the effect of rapamycin treatment on the relative fold changes (2^−ΔΔ^*^CT^*) in the mRNA expression of *SREBP2*, *LDLR*, and *PCSK9*. (b and c) Bar diagrams showing the effect of rapamycin treatment on the (b) levels of cellular cholesterol and (c) absolute expression (2^−Δ^*^CT^*) of kDNA to measure parasitic load in RA-supplemented L. donovani-infected Mϕ and rapamycin-treated, RA-supplemented L. donovani-infected Mϕ. Their respective levels in Mϕ only and L. donovani-infected Mϕ are shown with horizontal dotted lines. The experiments were performed in three independent sets, and each set was performed in triplicate. The difference between two conditions is considered significant if the *P* value is ≤0.05.

Our earlier findings confirmed that RA increased the cellular cholesterol and is not mediated through mTOR pathway (9). Moreover, we had observed increased mRNA expression of *RAPTOR*, possibly indicating the effective assembly of mTORC1, upon RA supplementation of L. donovani-infected Mϕ. Intrigued by this, we measured the expression of genes involved in the pathway under similar conditions along with an mTORC1 pathway inhibitor, i.e., rapamycin. First, we detected the mRNA expression of genes involved in cholesterol biosynthesis and uptake, and we found unaltered or marginally increased mRNA expression of *SREBP2* and *LDLR* in RA-supplemented L. donovani-infected cells upon treatment with rapamycin. Rapamycin treatment did not cause any substantial change in the expression of *PCSK9* mRNA in RA-supplemented L. donovani-infected Mϕ (Fig. 5a). This suggests that mTORC1 may not be involved in the RA-mediated increase of cellular cholesterol levels in L. donovani-infected Mϕ. To confirm this, we measured cellular cholesterol levels and parasite loads upon rapamycin treatment of RA-supplemented and L. donovani-infected Mϕ. Findings showed increased cholesterol levels (Fig. 5b) and decreased parasite loads under this condition (Fig. 5c).

## DISCUSSION

L. donovani, as it cannot synthesize cholesterol *de novo*, utilizes host cholesterol for early establishment of infection and impairing the immune response (10). Cholesterol is found at low levels in VL patients (11) and is a key component of the eukaryotic plasma membrane that regulates structural stability, fluidity, and maintenance (12). Its concentration in extrahepatic cells (i.e., Mϕ) is tightly regulated in the cell as well as in subcellular organelles. It is either endogenously synthesized inside the cells from acetyl-CoA using a complex pathway regulated by HMG-CoA reductase or acquired from endocytosing LDL through LDLR expressed on the surfaces of cells. Few proteins, such as NPC-1 and NPC-2, are required to capture cholesterol in the lysosome and subsequently transfer it to the other side of membrane (13). It can also be secreted in the form of HDL through the ABCA1 surface transporter (14). In our findings, it is evident that L. donovani infection impairs the synthesis (HMGCR), uptake (LDLR), and efflux (ABCA1) of cellular cholesterol in infected Mϕ. Many of the genes encoding these regulatory proteins/receptors/transporters are transcriptionally governed by SREBP2, which is sequestered in the endoplasmic reticulum (ER). This protein senses the levels of cholesterol, and its expression increases in a feedback manner. Thereafter, it is released from the ER and transported to the nucleus for expression of various genes, including *LDLR*, etc. (15). We have shown here that L. donovani infection reduces this significant transcription factor in Mϕ, which possibly impairs the expression of *HMGCR*, *LDLR*, and *ABCA1*. We have already shown that the L. donovani infection decreases the levels of cellular cholesterol in Mϕ by impairing the mRNA expression of *npc-1* and *npc-2* (6). In the same study, we showed improvement in the levels of cellular cholesterol in L. donovani-infected Mϕ upon RA treatment, which resulted in reduced parasite burden (6). In another part of our study in healthy uninfected Mϕ, RA was shown to restore the levels of cellular cholesterol in an mTOR-independent manner (9).

In general, under cholesterol-depleted conditions, mTOR, which is a key regulator of cell growth, proliferation, and immune response, is activated. mTOR complex 1 (mTORC1), one of two forms of the complex in mammalian cells, assists in regulating cholesterol synthesis along with several other cellular processes, such as autophagy, protein synthesis through eukaryotic translation initiation factor 4E-binding protein 1 (4E-BP1), p70S6 kinase (p70S6K) activation, etc. (16). In the presence of nutrients and growth factors, Akt/PKB, PIP3 kinase signaling pathways, etc., activate mTORC1. Expression of *RAPTOR*, encoding an important regulatory protein of mTORC1, is also controlled and poses an additional regulatory step in addition to phosphorylation in the functioning of mTORC1 proteins. In *Leishmania* infection, the status of the mTOR pathway is not clear. One report suggests increased phosphorylation of serine 2148 and 2481 residues in the mTOR kinase domain (7); however, another report suggests that inactivation of the mTOR pathway due to cleavage of the mTOR complex and activation of 4E-BP1 supports proliferation of *Leishmania* (17). We believe that assembly of various proteins in the complex is a first as well as a prerequisite step for the functioning of mTORC1 pathway prior to phosphorylation of serine residues in the kinase domain. Hence, we measured the expression of *RAPTOR* in L. donovani-infected cells and found a significant decrease in its mRNA expression. An intact as well as activated mTORC1 induces the release and expression of SREBP2 protein, which in turn activates the expression of various genes involved in cholesterol biosynthesis and uptake. This explains the decrease in the mRNA expression of *LDLR* in L. donovani-infected Mϕ, which is in accordance with the findings of low cholesterol in L. donovani-infected Mϕ.

It has always been of utmost importance to look for a substance, preferably a micronutrient, which potentiates the immune function of infected Mϕ. RA, in our earlier findings, showed an increase in the levels of cellular cholesterol. More importantly, RA overcomes the L. donovani-driven loss of cellular cholesterol and reverts it to optimum levels. Additionally, in the present study, we found that L. donovani infection compromises the expression of *SREBP2*, possibly resulting in reduced expression of *HMGCR*, *ABCA1*, and *LDLR.* This suggests impaired cholesterol biosynthesis and uptake.

In summary, RA entry into the infected cell is not affected, and it restores the immune response. RA increases the levels of cellular cholesterol in Mϕ, which is mTOR independent, as rapamycin abrogates the RA-driven increase in cellular cholesterol (9). However, in L. donovani-infected cells, inhibition of mTORC1 by rapamycin did not reverse the RA-mediated increase in expression of *SREBP2* and *LDLR* mRNA and thus the cellular cholesterol. This suggests that RA, in L. donovani-infected Mϕ, stimulates the mRNA expression of these genes involved in cholesterol synthesis and uptake in an mTOR-independent manner. One possibility which cannot be excluded is that RA binds to RAR-RXR and directly activates the expression of genes in an mTOR-independent manner. To corroborate this possibility, we explored the presence of RARE sites and/or binding of RAR-RXR complex upstream of genes such as *SREBP2* and *LDLR*. It has been reported that retinoic acid receptor binds to the rs57217136 domain of *LDLR* (18). Furthermore, mRNA expression of *SREBP2* and *LDLR* increased upon all-*trans*-retinoic acid (ATRA) treatment in MCF-7 cells (19). Thus, direct binding of RA through the RAR-RXR complex cannot be ruled out.

## MATERIALS AND METHODS

### Culture of L. donovani promastigotes and infection of J774A.1 cells.

L. donovani promastigotes (DD8 strain) were cultured in M199 medium (catalog no. 31100035; Gibco Life Technologies, USA) supplemented with 10% heat-inactivated fetal bovine serum (FBS) (catalog no. 10270106; Gibco Life Technologies, USA) and 10 mL/L of an antibiotic cocktail containing penicillin and streptomycin (Pen-Strep; catalog no. A018-5X100ML; Himedia, India). The culture was regularly maintained in a 25°C incubator. J774A.1 mouse Mϕ were maintained at 37°C and 5% CO_2_ in RPMI 1640 media (catalog no. 31800-022; Gibco Life Technologies, USA) with 10 mL/L of Pen-Strep and 10% heat-inactivated FBS (6).

After 1 × 10^6^ Mϕ were allowed to adhere in each well of the culture plate for 6 h in a 5% CO_2_ incubator at 37°C, nonadherent cells were removed and L. donovani promastigotes were added to adherent Mϕ for 12 h at a 10:1 (L. donovani to Mϕ) ratio. Once infection was established, unbound parasites were washed off with incomplete medium. Upon completion of the experiment, the parasite load was measured quantitatively using kDNA gene expression (6).

### Treatment with RA and rapamycin.

Before RA supplementation, infected cells were treated with 50 nM rapamycin (catalog no. R0097; TCI, Japan) for 2 h (20). Subsequently, 30 μg/mL of RA (equivalent to 100 μM; ATRA) (catalog no. R2625; Sigma-Aldrich, USA) was given for 12 h as per the experimental layout (21). This nonphysiological dose of RA was purposely chosen to observe its therapeutic effect on Mϕ. Following the completion of experiments, cells were harvested for RNA and genomic DNA (gDNA) isolation.

### RNA and cDNA preparation.

Total RNA was isolated using TRIzol (catalog no. 9108; TaKaRa Bio, USA) by adding 1 mL to harvested cells for a minimum period of 1 to 2 h. Subsequently, chloroform was added in a 10:2 (TRIzol to chloroform) ratio, mixed properly, and incubated for 10 min at room temperature. The suspension was centrifuged at 21,952 × *g* for 20 min at 4°C. After centrifugation, the uppermost layer containing RNA was transferred to a fresh microcentrifuge tube, and 500 μL of isopropanol was added. The tube was kept at room temperature for 10 min and then centrifuged at 21,952 × *g* for 20 min at 4°C. The precipitated RNA pellet was washed with 75% ethanol at least twice at 6,300 × *g* for 5 min at 4°C. After that, the RNA pellet was resuspended in 20 μL of nuclease-free water (catalog no. ML064; Himedia, India) (22).

RNA was quantified using a Picogene Nanodrop instrument, and 1 μg RNA was used to prepare cDNA. For cDNA preparation, 1 μL oligo(dT) (catalog no. S0131; Thermo Fisher Scientific, USA) primers, 1 U/μL murine leukemia virus (MuLV) reverse transcriptase (RT) (catalog no. M0253S; GeneAmp kit; Roche, Basel, Switzerland), 5 mM MuLV RT buffer, 1.25 mM deoxyribonucleoside triphosphates (dNTPs), 40 U/μL Ribolock (Thermo Fisher Scientific, USA), 10 mM dithiothreitol (DTT), and nuclease-free water in a final reaction volume of 20 μL were combined. The reaction mixture was kept in a thermal cycler under the following conditions: 95°C for 1 min followed by 42°C for 1 h and 72°C for 15 min (22).

### Real-time PCR.

Real-time amplification was performed using SYBR green master mix (catalog no. F416L; Thermo Fisher Scientific, USA) in a PikoReal real-time PCR system (catalog no. TCR0096; Thermo Fisher Scientific, USA). The primers were as follows: *SREBP2*, 5′-TCTGGACTTGATAGACGCA-3′ (forward primer [FP]), 5′-GAACAATGAACAAGGCTTAG-3′ (reverse primer [RP]); *RAPTOR*, 5′-CCTCACAAGTCTGATGCGGA-3′ (FP), 5′-TTCTCATCTGGCAAGGGCAG-3′ (RP); *LDLR*, 5′-GGTGGGACTTGGAAGAATAC-3′ (FP), 5′-CCCTTGTGTCTTGAGTAGTG-3′ (RP); *PCSK9*, 5′-GGAGAACCATACAGGACTTACC-3′ (FP), 5′-GCCAGCCACTCTGAATTTATG-3′ (RP); *HMGCR*, 5′-AGATAGGAACCGTGGGTGGT-3′ (FP), 5′-ACAAGATGTCCTGCTGCCAA-3′ (RP); *ABCA1*, 5′-GATGCGTCTGACCTTTGGGA-3′ (FP), 5′-CGGATGACATTGAGCACTGG-3′ (RP); *HGPRT*, 5′-GTTGGGCTTACCTCACTGCT-3′ (FP), 5′-TAATCACGACGCTGGGACTG-3′ (RP). The data were analyzed using the 2^−ΔΔ^*^cT^* method with *HGPRT* as the internal reference (6).

### Parasitic load assessment.

Tris-saturated phenol (catalog no. 12692; Sisco Research Laboratories Pvt. Ltd., India) was used to isolate gDNA. The L. donovani parasitic load was measured using SYBR green master mix, 5 pmol of each forward and reverse L. donovani-specific kDNA primer, and 1 μL of gDNA. Thermal cycling parameters were 95°C for 10 min and 40 cycles at 95°C for 60 s and 61°C for 15 s in a PikoReal real-time PCR system. The kDNA primers used in PCR were 5′-CTTTTCTGGTCCTCCGGGTAGG-3′ (FP) and 5′-CCACCCGGCCCTATTTTACACCAA-3′ (RP). Absolute expression (2^−Δ^*^cT^*) was calculated to measure the parasitic load in infected Mϕ by amplifying the L. donovani-specific kDNA copy number in isolated gDNA using quantitative PCR (qPCR) (23).

### HPLC-based detection of RA.

Culture supernatants were diluted in high-performance liquid chromatography (HPLC)-grade methanol in equal volumes and centrifuged at 2,800 × *g*. Clear supernatant was collected in autoclaved microcentrifuge tubes. RA was detected using an Agilent 1260 Infinity HPLC instrument. Chromatographic separation of the target molecule was carried out on X Bridge C_18_ columns (4.6 mm by 250 mm) as per the method described by Rahmayuni et al. (24), with some modifications. A 20-μL sample volume was used for each sample. The mobile phases solvent A (acetonitrile) and solvent B (HPLC water [0.1% formic acid]) were used as gradients for a 30-min run at a flow rate of 1.2 mL/min. The gradient flow was as follows: at 0 min, 8% solvent A; at 5 min, 8% solvent A; at 16 min, 50% solvent A; at 23 min, 95% solvent A; and at 30 min, 8% solvent A. A variable-wavelength detector (VWD) was used, and wavelength was set to 321 nm.

### Cellular cholesterol estimation.

Cholesterol from harvested cells was extracted using hexane-isopropanol and was subjected to biochemical estimation. First, cells were treated with hexane-isopropanol [3:2] for 30 min at room temperature, and the suspension was dried by sparging with nitrogen gas. Then, in the dried sample, 50 μL of isopropanol was added, followed by light vortexing. After that, 500 μL of cholesterol reagent (containing ≥200 IU/L cholesterol esterase, ≥150 IU/L cholesterol oxidase, ≥2,000 IU/L peroxidase, 20 mmol/L sodium phenolate, 0.5 mmol/L 4-aminoantipyrine, and 68 mmol/L phosphate buffer at pH 6.5) was added and incubated for 2 h at 37°C. Finally, the absorbance was measured at 505 nm (6, 25). Cholesterol concentration (in milligrams per deciliter) was calculated as (Δ*A*_sam_/Δ*A*_std_) × *C*_std_, where Δ*A*_sam_ is absorbance of the sample, Δ*A*_std_ is absorbance of the standard against the reagent blank, and *C*_std_ is the standard concentration (200 mg/dL).

### Confocal laser scanning microscopy.

We performed confocal laser scanning microscopy (Olympus, FV1000) to image and examine the cellular cholesterol levels using filipin III (catalog no. SC-205323A; Santa Cruz Biotechnology, USA). J774A.1 Mϕ were allowed to adhere to 12- to 15-mm coverslips (catalog no. TCP017-1X200NO; Himedia, India). Macrophages were infected with L. donovani for 12 h. As per the experimental layout, cells were treated with rapamycin and RA postinfection. Later, the slides were stained with 50 μg/mL filipin III (6, 25). Thereafter, the excess dye was removed by washing with 1× phosphate-buffered saline (PBS). Coverslips were mounted in glycerol mounting medium with an antifading agent (catalog no. P10144; Thermo Fisher Scientific, USA).

### Statistical analysis.

Experiments were independently repeated at least three times, and every experiment had at least two samples per group. Data were analyzed using GraphPad Prism 7 (GraphPad Prism, Inc., La Jolla, CA). Statistical differences were calculated using the paired *t* test and Wilcoxon signed ranked test or the unpaired *t* test and Mann-Whitney test; *P* values of ≤0.05 were considered significant.
